# Development and validation of an ultrasound-based prediction model for differentiating between malignant and benign solid pancreatic lesions

**DOI:** 10.1007/s00330-022-08930-0

**Published:** 2022-06-25

**Authors:** Jiayan Huang, Jie Yang, Jianming Ding, Jing Zhou, Rui Yang, Jiawu Li, Yan Luo, Qiang Lu

**Affiliations:** 1 Department of Ultrasound, West China Hospital of Sichuan University, Chengdu, 610041 China; 2 Department of Ultrasound, Tianjin Third Central Hospital, Tianjin, 300170 China; 3 Department of Ultrasound, The Affiliated Hospital of Southwest Medical University, Luzhou, 646000 Sichuan China; 4 Department of Ultrasound, Laboratory of Ultrasound Medicine, West China Hospital of Sichuan University, Chengdu, 610041 China

**Keywords:** Pancreatic neoplasms, Ultrasonography, Contrast media, Nomograms

## Abstract

**Objective:**

To identify the diagnostic ability of precontrast and contrast-enhanced ultrasound (CEUS) in differentiating between malignant and benign solid pancreatic lesions (MSPLs and BSPLs) and to develop an easy-to-use diagnostic nomogram.

**Materials and methods:**

This study was approved by the institutional review board. Patients with pathologically confirmed solid pancreatic lesions were enrolled from one tertiary medical centre from March 2011 to June 2021 and in two tertiary institutions between January 2015 and June 2021. A prediction nomogram model was established in the training set by using precontrast US and CEUS imaging features that were independently associated with MSPLs. The performance of the prediction model was further externally validated.

**Results:**

A total of 155 patients (mean age, 55 ± 14.6 years, M/F = 84/71) and 78 patients (mean age, 59 ± 13.4 years, M/F = 36/42) were included in the training and validation cohorts, respectively. In the training set, an ill-defined border and dilated main pancreatic duct on precontrast ultrasound, CEUS patterns of hypoenhancement in both the arterial and venous phases of CEUS, and hyperenhancement/isoenhancement followed by washout were independently associated with MSPLs. The prediction nomogram model developed with the aforementioned variables showed good performance in differentiating MSPLs from BSPLs with an area under the curve (AUC) of 0.938 in the training set and 0.906 in the validation set.

**Conclusion:**

Hypoenhancement in all phases, hyperenhancement/isoenhancement followed by washout on CEUS, an ill-defined border, and a dilated main pancreatic duct were independent risk factors for MSPLs. The nomogram constructed based on these predictors can be used to diagnose MSPLs.

**Key Points:**

*• An ill-defined border and dilated main pancreatic duct on precontrast ultrasound, hypoenhancement in all phases of CEUS, and hyperenhancement/isoenhancement followed by washout were independently associated with MSPLs.*

*• The ultrasound-based prediction model showed good performance in differentiating MSPLs from BSPLs with an AUC of 0.938 in the training set and 0.906 in the external validation set.*

*• An ultrasound-based nomogram is an easy-to-use tool to differentiate between MSPLs and BSPLs with high efficacy.*

**Supplementary Information:**

The online version contains supplementary material available at 10.1007/s00330-022-08930-0.

## Introduction

Pancreatic cancer is currently the 4th leading cause of cancer-related death and continues to increase in incidence in both men and women [1]. Solid pancreatic lesions (SPLs) are a common abnormality found in both symptomatic and asymptomatic patients, with pancreatic carcinoma accounting for the highest proportion of such lesions, and has an incidence ranging from 31 to 34% [2]. Imaging modalities, including transabdominal ultrasound (US), computed tomography (CT), magnetic resonance imaging (MRI), endoscopic ultrasound (EUS), and positron emission tomography (PET), are commonly used in the diagnosis of pancreatic cancer. However, some inherent drawbacks limit the use of the aforementioned modalities for the diagnosis of pancreatic diseases. For instance, EUS is an invasive tool, and PET frequently has difficulty distinguishing pancreatitis from pancreatic cancer [3]. Although CT and MRI have the capability for disease staging and assessing the resectability of pancreatic cancer [4], radiation exposure and iodine allergy in CT as well as renal function-dependence of the patient and the lower spatial resolution of MRI likewise restrict the application of these modalities in specific cases. Conventional US is commonly used as a screening tool in the detection and initial assessment of pancreatic lesions, but the diagnostic performance of US reported in the literature for pancreatic cancer varies, with sensitivity and specificity values ranging from 68 to 98% and from 50 to 100%, respectively [5–7]. Currently, with the application of contrast agents and low mechanical index real-time harmonic imaging, contrast-enhanced ultrasound (CEUS) has been reported to significantly improve the efficacy of US in characterising suspicious pancreatic lesions [8–12] and is recommended for the diagnosis of SPL by guidelines [1, 13]. The main advantage of CEUS over other imaging modalities is the high temporal resolution which allows for real-time evaluation of the pancreas. Moreover, CEUS has the highest contrast resolution of any clinical imaging modality [14]. The ultrasound contrast agent (SonoVue) is a pure blood pool agent that allows CEUS to truly reflect the microvascular perfusion of tumours because it does not enter the extracellular space.

In clinical practice, the accurate identification of benign and malignant SPLs is crucial as the diagnosis may change the treatment strategy for a patient and help avoid unnecessary biopsy or even surgery. To the best of our knowledge, the majority of studies focusing on the diagnosis of solid pancreatic lesions by using CEUS have mainly concentrated on several specific pathologies and were single-centre investigations that did not propose an explicit diagnostic criterion for the differential diagnosis between malignant and benign SPLs.

Herein, we conducted a multicentre study to evaluate the diagnostic ability of transabdominal CEUS to differentiate between malignant and benign SPLs by developing and validating an easy-to-use diagnostic nomogram model based on precontrast US and CEUS.

## Materials and methods

### Patients

This retrospective study was approved by the institutional review board, and the requirement for informed consent was waived. Patients were enrolled from three centres. From March 2011 to June 2021, 155 SPL patients with pathology results were consecutively collected from one tertiary medical centre and constituted the training cohort, which included 95 malignant solid pancreatic lesions (MSPLs) (62 men and 33 women; mean age, 58.7 ± 9.3 years) and 60 benign solid pancreatic lesions (BSPLs) (22 men and 38 women; mean age, 43.4 ± 16.5 years). Additionally, 78 patients with pathologically confirmed SPLs (47 MSPLs and 31 BSPLs) between January 2015 and June 2021 from two other tertiary hospitals comprised the external validation set (Table 1). Pathology distribution of tumours in the training and validation sets is also displayed in Table 1. Specifically, neuroendocrine neoplasms (NETs) with a pathological differentiation of grade 1 were designated as BSPLs, whereas grade 2 and 3 NETs were considered MSPLs.

**Table 1:** Patient demographics and distribution of tumours in the training and validation sets

	Training set			Validation set		
	MSPL (95)	BSPL (60)		MSPL (47)	BSPL (31)	
Patients			*p* value			*p* value
Sex (M/F)	62/33	22/38	< .001	27/20	9/22	.03
Age (year)	58.7 ± 9.3	43.4 ± 16.5	< .001	61.1 ± 0.6	49.2 ± 14.2	< .001
Pathology distribution of SPL	PDAC (85)	G1 NET (20)		PDAC (39)	G1 NET (15)	
	M (3)	SPT (16)		M (5)	SPT (6)	
	OM (7)	MFCP (6)		OM (3)	MFCP (4)	
		OB (18)			OB (6)	

Data in the parentheses are numbers of patients or pancreatic nodules. *MSPL* malignant solid pancreatic lesion, *BSPL* benign solid pancreatic lesion, *PDAC* pancreatic ductal carcinoma, *M* metastasis, *OM* other types of malignancy, *G1 NET* neuroendocrine tumour with grade 1 pathological differentiation, *SPT* solid pseudopapillary tumour, *MFCP* mass-forming chronic pancreatitis, *OB* other types of benign lesion

**Fig. 1:**
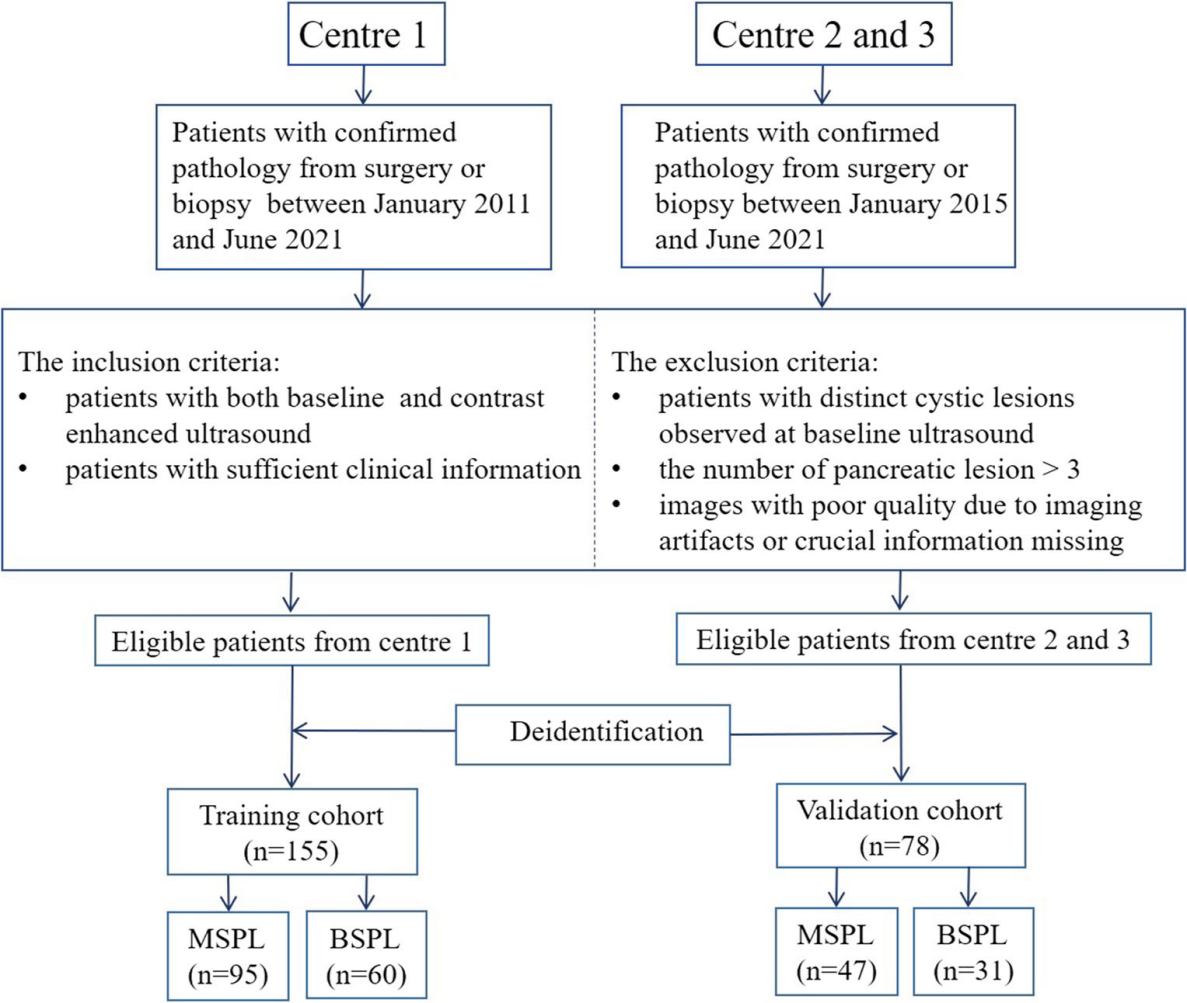
Patient selection flowchart. MSPL, malignant solid pancreatic lesion, BSPL, benign solid pancreatic lesion

The inclusion criteria were as follows: (I) definite histopathological results either from surgery or biopsy; (II) both baseline US and CEUS of the target SPL; and (III) sufficient clinical data, including demographics and laboratory results. The exclusion criteria were as follows: (I) distinct cystic lesions observed on baseline ultrasound images; (II) more than one SPL; and (III) poor image quality due to imaging artefacts or missing crucial information. After deidentification of the patients’ information, images of the SPLs were randomly numbered as independent files for further evaluation. The patient selection flowchart is presented in Fig. 1.

### Baseline ultrasound and CEUS

All enrolled patients underwent baseline ultrasound and CEUS examinations using a Philips IU 22 or EPIQ7 ultrasound system (Philips Healthcare) equipped with a C5-1 probe or a Mindray Resona 7 ultrasound system (Mindray Medical Solutions) mounted with an SC6-1 transducer. CEUS was performed after baseline ultrasound scanning of the pancreas. A bolus injection of 1.2–2.4 mL SonoVue (Bracco) was given via a 20-gauge angiocatheter needle placed in the antecubital vein followed by a 5 mL flush of 0.9% sodium chloride solution. The imaging timer was initiated when the SonoVue injection was complete. Still images and video clips from the baseline ultrasound and CEUS examinations were digitally stored for further evaluation.

### Image analysis

The preoperative ultrasound images of the enrolled patients were processed as individual files and numbered randomly after deidentification by a radiologist (J.Y.). Then, two radiologists (Q.L. and J.W.L., with more than 14 and 6 years of experience in abdominal CEUS examination, respectively) who were blinded to clinical information and pathological results reviewed the images and recorded their judgments independently. Specifically, the following imaging features were evaluated: (a) maximum SPL diameter; (b) SPL location (head or body/tail of the pancreas); (c) tumour border (clear or ill-defined); (d) shape (irregular or regular); (e) echogenicity (hyperechoic, isoechoic, or hypoechoic); (f) diameter of the main pancreatic duct (MPD) (≥ 4 mm or < 4 mm); (g) diameter of the common bile duct (CBD) (≥ 10 mm or < 10 mm); (h) adjacent vessel involvement (including the main portal vein, superior mesenteric artery and vein, celiac axis, splenic vein, and common hepatic artery); (i) localised pancreatic swelling; (j) necrotic contents within the SPL; (k) calcification within the SPL; and (l) enhancement degree of the SPL in the arterial and venous phases (hyperenhancement, isoenhancement, hypoenhancement, or no enhancement). Interobserver agreement on the baseline US and CEUS imaging features was evaluated by the Kappa value. When there were discordant results, the final judgement was obtained by consensus after further data analysis. The training set in the current study referred to the data (features) extracted from the SPLs on baseline US and CEUS imaging in patients from one of the three centres. The training data set was used to develop a prediction model based on statistically significant predictors for malignant SPLs. The performance of the prediction model was then assessed in the external validation data set (data from the other two centres)

### Statistical analysis

Quantitative data are presented as the mean ± standard deviation, and qualitative data are presented as absolute numbers and percentages. Student’s *t* test or the Mann–Whitney *U* test was used for continuous variables, and the *χ*^2^ or Fisher exact test was used for categorical variables. Univariate logistic regression analysis was used to identify features associated with MSPL, and multivariate logistic regression analysis was used to develop a prediction model based on the optimal features for diagnosing of MSPL. The Hosmer–Lemeshow test was used to determine the goodness of fit of the logistic regression model. A nomogram was built based on this prediction model [15]. The variable having the greatest impact in this model was assigned 100 points, and the other variables were then scored accordingly depending on their effect relative to the parameter with the greatest effect. The diagnostic efficacy of the prediction model in the training and validation sets was evaluated with the area under the receiver operating characteristic curve (AUC).

Interobserver agreement in analysing the imaging features of SPL was evaluated with Cohen’s kappa coefficient. A *κ* value < 0.2 indicates poor agreement; 0.2–0.4 indicates fair agreement; 0.41–0.6 indicates moderate agreement; 0.61–0.8 indicates good agreement; and 0.8–1 indicates almost perfect agreement. Significance was defined as *p* < 0.05, except in univariate logistic analysis where *p* < 0.1 was considered to be significant. All statistical analyses were performed using a software package (STATA 15.0, Stata Corporation).

## Results

### Clinical and pathologic characteristics

The patient demographics and pathological distribution of the tumours in the training and validation sets are summarised in Table 1. There were significant differences in regard to patients’ sex and age. Moreover, significant differences were found in serum biomarkers in the training and validation cohorts between patients with MSPLs and BSPLs (Supplementary Table). The median time between CEUS and surgery was 8.2 days (range, 1–48 days).

### Precontrast and CEUS features of the training set

The imaging characteristics of MSPLs and BSPLs were analysed and compared in the training set (Table 2). On baseline ultrasound, significant differences were found between the two entities regarding tumour size, dilation of the MPD and CBD, border and shape of the lesion, and adjacent vessel involvement. On CEUS imaging, the SPLs demonstrated five enhancement modes, namely hypoenhancement in both the arterial phase (AP) and venous phase (VP), hyperenhancement in both the AP and VP, iso- or hyperenhancement in the AP followed by hypoenhancement in the VP, hyper- or isoenhancement in the AP followed by isoenhancement in the VP, and no enhancement in any phase. Hypoenhancement in both the AP and VP was identified in 75.8% (72/95) of MSPLs, which was more frequent than in 16.7% (10/60) of BSPLs (*p* < .001). There was a higher proportion of lesions without washout in the BSPL group than that in the MSPL group (55% (33/60) vs. 33.7% (32/95), *p* < .014).

**Table 2:** Comparison of imaging characteristics of MSPL and BSPL in the training set

Imaging characteristics	MSPL (*n* = 95)	BSPL (*n* = 60)	*p* value
Baseline ultrasound			
Nodule size (cm)	3.7 ± 1.5	3.1 ± 1.6	.02
Location			.52
Head	64 (67.4)	45 (75)	
Body or tail	31 (32.6)	15 (25)	
Echo			.71
Hypo-/iso-	94 (98.9)	58 (96.7)	
Hyper	1 (1.1)	2 (3.3)	
MPD dilation (≥ 4 mm)	47 (49.5)	7 (11.7)	< .001
CBD dilation (≥ 10 mm)	21 (22.1)	2 (3.3)	.003
Ill-defined lesion border	89 (93.7)	20 (33.3)	< .001
Irregular lesion shape	89 (93.7)	22 (36.7)	< .001
Adjacent vessel involvement	17 (17.9)	2 (3.3)	.01
Necrotic contents	11 (11.6)	7 (11.7)	.80
Calcification	2 (2.1)	3 (5)	.59
CEUS manifestations			
Arterial phase			< .001
Hyperenhancement	16 (16.8)	34 (56.7)	
Isoenhancement	7 (7.4)	14 (23.3)	
Hypoenhancement	72 (75.8)	10 (16.7)	
No enhancement	0 (0)	2 (3.3)	
Venous phase			< .001
Hyperenhancement	2 (2.1)	15 (25)	
Isoenhancement	2 (2.1)	21 (35)	
Hypoenhancement	91 (95.8)	22 (36.7)	
No enhancement	0 (0)	2 (2.1)	
CEUS enhancement pattern†			< .001
Pattern A	4 (4.2)	38 (63.3)	
Pattern B	72 (75.8)	10 (16.7)	
Pattern C	19 (20)	12 (20)	

Except for nodule size, data are pancreatic nodules and data in parentheses are percentages. Qualitative data are presented as numbers and percentage, quantitative data as mean ± standard deviation. *MSPL* malignant solid pancreatic lesion, *BSPL* benign solid pancreatic lesion, *MPD* main pancreatic duct, *CBD* common bile duct, *CEUS* contrast-enhanced ultrasound, *AP* arterial phase, *VP* venous phase

†Five CEUS enhancement modes of solid pancreatic lesions were summarised as three patterns: pattern A: hyperenhancement in both the AP and VP, hyper- or isoenhancement in the AP followed by isoenhancement in the VP, or no enhancement through either phase; pattern B: hypoenhancement in both the AP and VP; and pattern C: iso- or hyperenhancement in the AP followed by hypoenhancement in the VP

### Development of the sonographic imaging prediction model

**Fig. 2:**
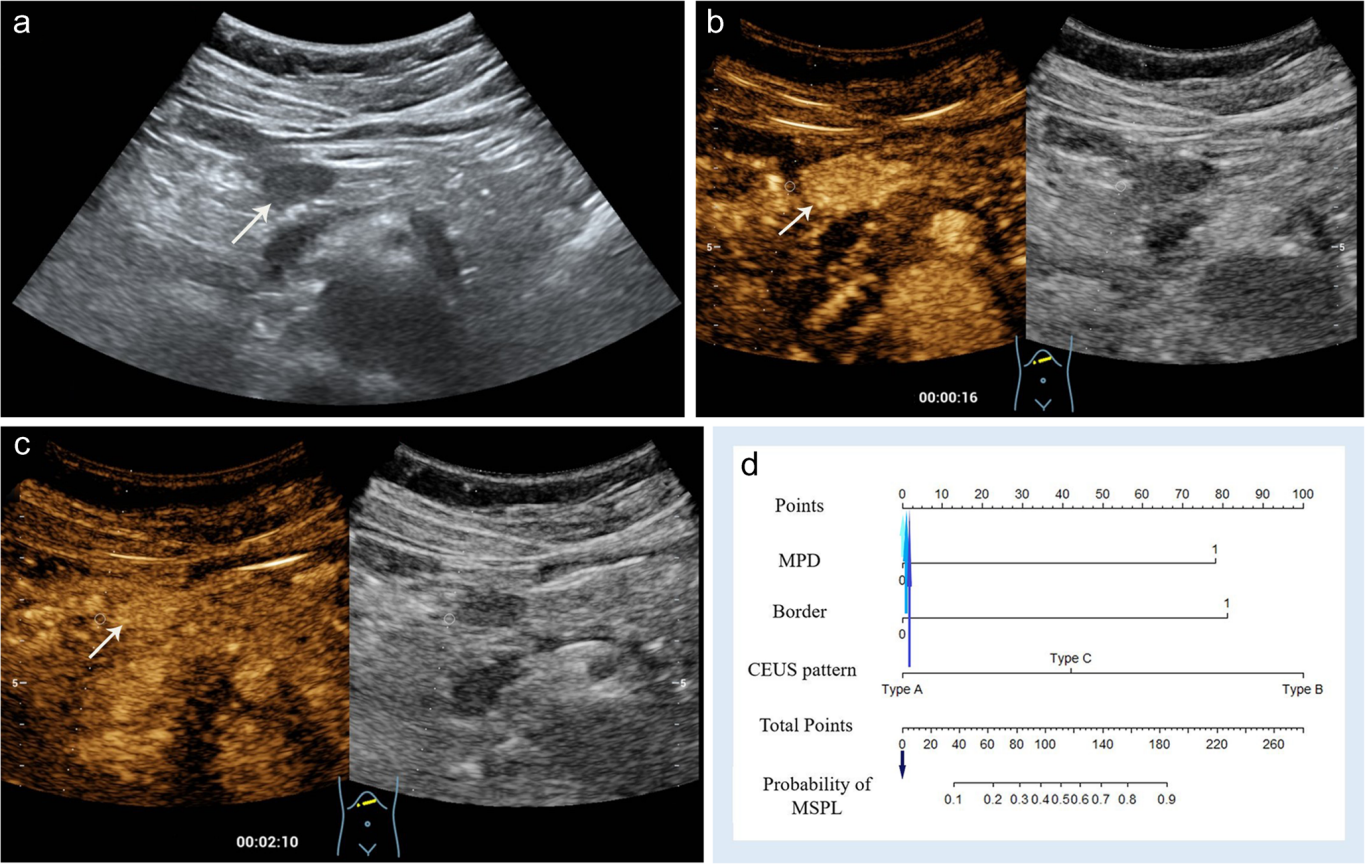
Contrast-enhanced US of a 51-year-old woman with a 2.0-cm hypoechoic solid lesion (arrow) in the neck of the pancreas. **a** The lesion (arrow) had a clear border, and the diameter of the main pancreatic duct was within the normal range. **b** Arterial phase hyperenhancement (arrow) was seen on contrast-enhanced US. **c** The lesion showed slight hyperenhancement (arrow) relative to the surrounding pancreatic parenchyma in the venous phase. **d** The lesion received a score of 0 according to the nomogram, corresponding to a less than 10% probability of malignancy. A neuroendocrine tumour with pathological differentiation of grade 1 was confirmed by pathological analysis

**Fig. 3:**
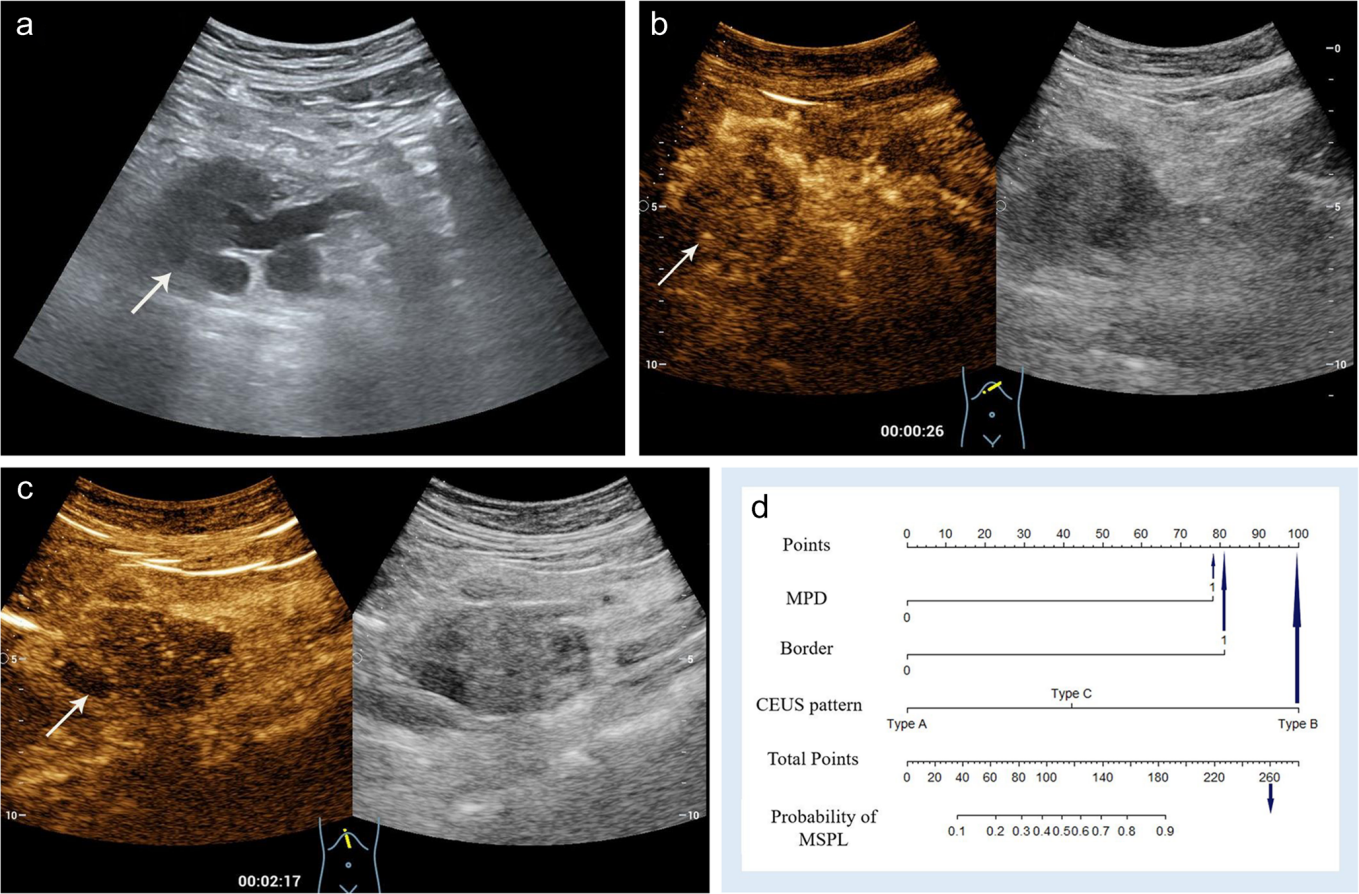
Contrast-enhanced US of a 60-year-old woman with a 5.3-cm hypoechoic solid lesion in the head of the pancreas. **a** The lesion showed an ill-defined border (arrow) and dilated main pancreatic duct measuring 9 mm. **b**, **c** Hypoenhancement of the tumour was demonstrated in both the arterial phase (**b**) and venous phases (**c**). **d** A total of 260 points were assigned to the lesion according to the nomogram, corresponding to a higher than 90% probability of being a malignant solid pancreatic lesion. Poorly differentiated pancreatic ductal adenocarcinoma was confirmed by histopathology

**Fig. 4:**
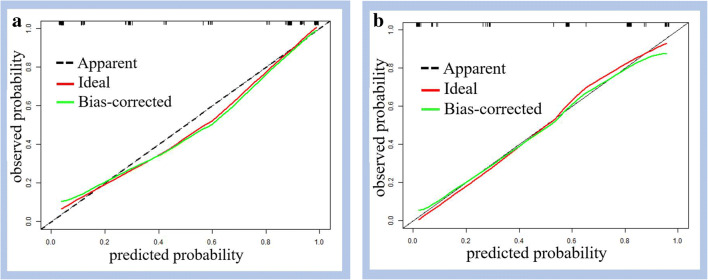
The predictive reliability and goodness of fit of the logistic regression were assessed by using the Hosmer–Lemeshow test, showing *p* values of 0.726 and 0.323 in the training (**a**) and validation (**b**) sets, respectively

A prediction model was then developed based on the imaging features that significantly differed between MSPLs and BSPLs on baseline US and CEUS in the training set (Table 3). The aforementioned five CEUS enhancement modes of SPLs were summarised as three patterns according to the enhancement phase and intensity relative to the surrounding parenchyma: pattern A: hyperenhancement in both the AP and VP (Fig. 2), hyper- or isoenhancement in the AP followed by isoenhancement in the VP, or no enhancement through either phase; pattern B: hypoenhancement in both the AP and VP (Fig. 3); and pattern C: iso- or hyperenhancement in the AP followed by hypoenhancement in the VP. Univariate analysis revealed that a dilated MPD and CBD, ill-defined border, adjacent vessel involvement, and CEUS enhancement pattern were significant risk factors for MSPL in the training set. Furthermore, a dilated MPD, ill-defined border, and CEUS enhancement pattern were independent risk factors for MSPL according to the multivariate analysis. A prediction model was then developed based on the results of the logistic analysis. The Hosmer–Lemeshow test showed good predictive reliability of prediction and goodness of fit for the logistic regression models with *p* values of 0.726 and 0.323 for the training and validation sets, respectively (Fig. 4).

**Table 3:** The univariate and multivariate analysis of patients with SPL in the training set

Imaging features	Univariate analysis			Multivariate analysis		
	OR	95% CI	*p* value	OR	95% CI	*p* value
Nodule size (cm)	1.20	0.97–1.49	.11	--	--	--
Location (head or body/tail)	0.67	0.34–1.31	.24	--	--	--
MPD dilation (≥ 4 mm)	14.98	5.59–52.31	< .001	9.37	2.59–43.05	.001
CBD dilation (≥ 10 mm)	16.21	3.25–294.59	.007	1.20	0.13–28.80	.89
Ill-defined border	28.83	11.98–77.37	< .001	10.77	3.42–37.78	< .001
Adjacent vessel involvement	11.75	2.31–214.80	.02	5.77	0.57–158.03	.20
Necrotic contents	1.88	0.68–6.06	.25	--	--	--
CEUS enhancement pattern†						
Pattern A	--	--	Reference	--	--	Reference
Pattern B	48.89	17.58–157.56	< .001	15.19	4.17–63.81	< .001
Pattern C	7.49	2.60–23.71	< .001	2.28	0.49–10.73	.29

Variables with *p* < 0.1 in the univariate analysis were included in the multivariate analysis

*OR* odd ratio, *CI* confidence intervals, *MPD* main pancreatic duct, *CBD* common bile duct, *CEUS* contrast-enhanced ultrasound, *AP* arterial phase, *VP* venous phase

†Pattern A: hyperenhancement in both the AP and VP, hyper- or isoenhancement in the AP followed by isoenhancement in the VP, or no enhancement through either phase; pattern B: hypoenhancement in both the AP and VP; and pattern C: iso- or hyperenhancement in the AP followed by hypoenhancement in the VP

### Development of the nomogram for the prediction model

**Fig. 5:**
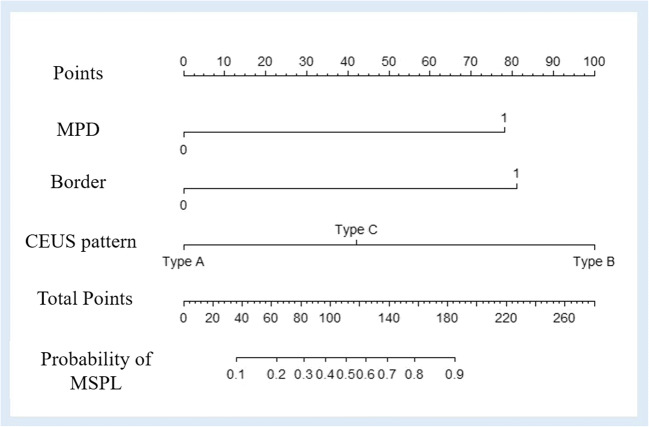
A sonography-based nomogram was developed in the primary cohort, incorporating dilated main pancreatic duct (MPD), ill-defined lesion border, and contrast-enhanced US (CEUS) patterns. Each variable was assigned corresponding predictor points from the point scale drawn at the top. The points of each variable were summed, and the total points are projected onto the bottom scale to determine the probability of malignant solid pancreatic lesions (MSPLs)

**Fig. 6:**
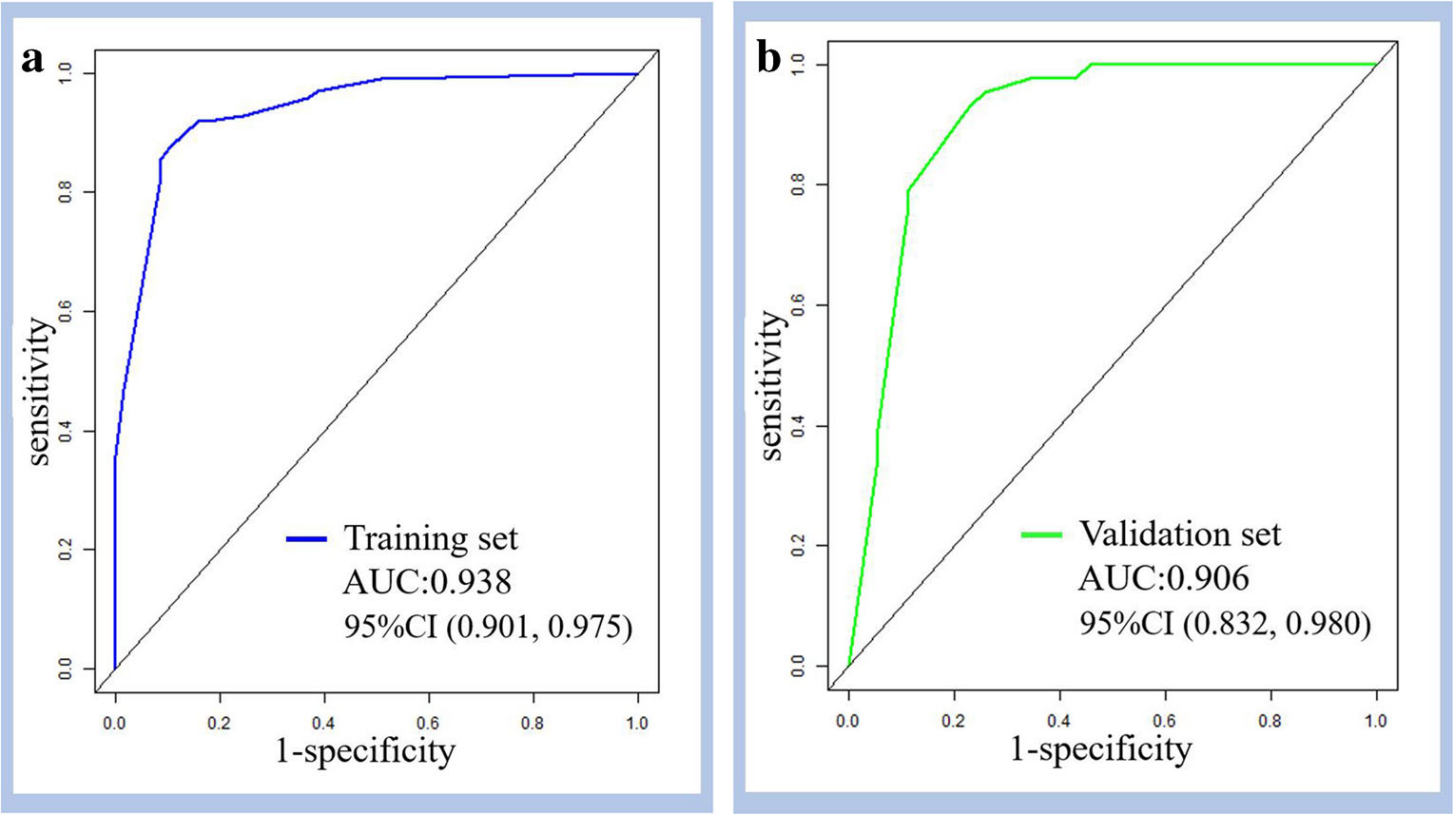
The receiver operating characteristic (ROC) curves of the sonography-based nomogram in the training (**a**) and validation (**b**) sets. AUC, area under the receiver operating characteristic curve, CI, confidence interval

A nomogram was constructed based on the prediction model derived in the training set (Fig. 5). CEUS pattern A was set as the reference category in the univariate and multivariate analyses. Accordingly, CEUS pattern B was assigned 100 points, because it had the greatest effect in the prediction model. CEUS pattern C, dilated MPD, and ill-defined SPL border were scored based on their effect proportional to that of CEUS pattern B. The discrimination efficacy was comparable between the training and validation sets, with AUCs of 0.938 (95% CI: 0.901–0.975) and 0.906 (95% CI: 0.832–0.980), respectively (Fig. 6).

## Discussion

Preoperative discrimination between malignant solid pancreatic lesions (MSPLs) and benign solid pancreatic lesions (BSPLs) is crucial for determining the treatment and prognosis of patients with suspicious solid pancreatic lesions (SPLs). In the present study, we proposed a nomogram model based on imaging features from baseline US and contrast-enhanced ultrasound (CEUS), with an AUC of 0.938 in the training set and 0.906 in the validation set, to differentiate between MSPLs and BSPLs in a simple and effective way.

The value of transabdominal US in diagnosing pancreatic cancer remains controversial due to its variable diagnostic sensitivity and specificity in previous studies [5–7, 16]. However, transabdominal US is still used as a first-line screening tool in clinical settings and considered a favourable modality for routine medical examinations of asymptomatic individuals [17]. Compared with baseline US, CEUS has been reported to significantly improve the efficacy of characterising SPLs [8–12] and is recommended for the diagnosis of SPLs by established guidelines [1, 13]. In the current study, hypoenhancement in both the arterial phase (AP) and venous phase (VP), hyperenhancement or isoenhancement in the AP followed by hypoenhancement in the VP, ill-defined border, and dilated MPD were found to be independent risk factors for MSPLs, which is consistent with the findings of previous studies [18–21]. D’ Onofrio et al reported that, by using the hypovascularised characteristic of pancreatic ductal adenocarcinoma (PDAC), the pooled sensitivity, specificity, and diagnostic odds ratio of CEUS were 0.89, 0.84, and 61.12, respectively [21]. However, previous studies have placed more emphasis on the use of a certain feature, particularly hypovascularity on CEUS. Although PDAC accounts for the highest proportion of SPLs [2], there are other pathologically malignant entities, such as metastasis, that may demonstrate isoenhancement or hyperenhancement in the AP followed by washout in the VP. In the present study, we used all the aforementioned independent risk factors to construct a nomogram model and validated its robust diagnostic ability.

Amongst all suspected pancreatic lesions, mass-forming chronic pancreatitis (MFCP) bears the brunt of differential diagnosis with PDAC due to its overlapping clinical symptoms (e.g. abdominal pain, weight loss, nausea, and jaundice) as well as imaging manifestations [22–25]. Both PDAC and MFCP frequently present as hypoechoic lesions on precontrast US, and they may also share similar enhancement pattern on CEUS [9, 26]. In our study, half of the MFCP lesions (5/10) manifested iso- or slight hyperenhancement in the AP followed by isoenhancement in the VP. Focal pancreatitis typically shows isoenhancement relative to the adjacent pancreatic parenchyma, which is characterised by ‘parenchymographic’ enhancement [9, 27]. However, in patients with long-standing chronic inflammatory processes, inhomogeneous hypoenhancement may also be observed. In the present study, three out of ten MFCP lesions displayed hypoenhancement in both the AP and VP, and the other two cases presented as isoenhancement in the AP followed by late washout; thus, these lesions were prone to be regarded as malignant lesions. This phenomenon could be partially explained by the presence of a higher proportion of fibrous content in chronic inflammation processes, which may lead to a more difficult differential diagnosis between PDAC and MFCP [28, 29]. However, the combination of contrast-enhanced patterns and morphologic characteristics may perform better. According to our nomogram, the average scores of MSPLs and pancreatitis were 196 and 106 points, which corresponded to 90% and 45% possibilities for an MSPL, respectively. Therefore, our prediction model could be an effective tool in differentiating mass-forming pancreatitis from MSPL.

To the best of our knowledge, this is the first study using a CEUS-based nomogram to distinguish BSPLs from MSPLs with external validation. The pathological types in the present study (including the training and validation sets) mainly comprised PDAC, followed by neuroendocrine tumours, solid pseudopapillary tumours, metastases, and other rare malignant or benign tumours, which is comparable with previous studies [12, 28, 29]. Moreover, the nomogram model is simple to use and has high diagnostic efficiency and thus, it can facilitate the application of CEUS in the diagnosis of focal pancreatic lesions. Although various prediction models have been developed by studies using CT- or MRI-based radiomics and shown excellent sensitivity and specificity for the differentiation of specific pancreatic pathologies [30–34], the need for an accurate and easy-to-use method for the differentiation between MSPLs and BSPLs remains. CEUS is currently a widely used tool for pancreatic diseases and was deemed an effective tool for characterising pancreatic lesions and providing complementary diagnostic value to other imaging modalities. In a recent systematic review and meta-analysis, the investigators demonstrated that CEUS is a capable technique for characterising the enhancement pattern of benign and malignant pancreatic neoplasms [35]. Herein, we developed a CEUS-based nomogram, providing an easy-to-use tool for the differential diagnosis between MSPL and BSPL.

While encouraging, several limitations of our study need to be addressed. First, the number of some pathological types was small. For instance, the metastases and MFCP only composed a small portion of the SPLs. Therefore, studies with larger samples are needed to further validate the diagnostic power of the nomogram. Second, we only enrolled patients with SPLs. Further studies including both solid and cystic lesions may be needed for a more comprehensive evaluation of whether CEUS can be applied to assess focal pancreatic lesions. Last, as a retrospective study, selection bias may be inevitable, and prospective studies are required to further validate our results.

## Conclusion

In conclusion, hypoenhancement in both the AP and VP, hyper- or isoenhancement followed by washout on CEUS, ill-defined border, and dilated MPD were independent risk factors for MSPLs. The nomogram constructed based on these predictors can be used as an effective tool for the diagnosis of MSPLs.

## Supplementary information

ESM 1 (DOCX 20 kb)

## Abbreviations

AP: Arterial phase
AUC: Area under the curve
BSPL: Benign solid pancreatic lesion
CBD: Common bile duct
CEUS: Contrast-enhanced ultrasound
CT: Computed tomography
EUS: Endoscopic ultrasound
MFCP: Mass-forming chronic pancreatitis
MPD: Main pancreatic duct
MRI: Magnetic resonance imaging
MSPL: Malignant solid pancreatic lesion
PDAC: Pancreatic ductal adenocarcinoma
PET: Positron emission tomography
ROC: Receiver operating characteristic
SPL: Solid pancreatic lesion
US: Ultrasound
VP: Venous phase

## Declarations

### Guarantor

The scientific guarantor of this publication is Qiang Lu.

### Statistics and biometry

One of the authors (Jie Yang) has significant statistical expertise.

### Methodology

• retrospective

• diagnostic study

• multicentre study
