# Supplementary material for: Development and validation of an ultrasound-based prediction model for differentiating between malignant and benign solid pancreatic lesions
Source: Eur Radiol. 2022 Jun 25;32(12):8296–305. doi: 10.1007/s00330-022-08930-0 (PMC9705429; doi:10.1007/s00330-022-08930-0)
Supplement: Supplementary file 1 — (DOCX 20 kb) [file 330_2022_8930_MOESM1_ESM.docx]

| **Supplementary table1. Serum Biomarkers of Patients with SPL in the Training and Validation Sets** | | | | | | | |
| --- | --- | --- | --- | --- | --- | --- | --- |
| Clinical factors | Training set (n=155) | | |  | Validation set (n=78) | | |
|  | MSPL (n=95) | BSPL (n=60) | *p* value |  | MSPL (n=47) | BSPL (n=31) | *p* value |
| CEA (ng/mL) |  |  | <.001 |  |  |  | <.001 |
| <3.4 | 22 (25.3) | 32 (66.7) |  |  | 8 (18.2) | 19 (67.9) |  |
| > 3.4 | 65 (74.7) | 16 (33.3) |  |  | 36 (81.8) | 9 (32.1) |  |
| NA | 8 | 12 |  |  | 3 | 3 |  |
| CA 19-9 (U/mL) |  |  | <.001 |  |  |  | <.001 |
| <30 | 20 (22.5) | 46 (93.9) |  |  | 14 (31.8) | 26 (76.5) |  |
| >30 | 69 (77.5) | 3 (6.1) |  |  | 30 (68.2) | 2 (5.9) |  |
| NA | 6 | 11 |  |  | 3 | 3 |  |
| CA 125 (U/mL) |  |  | <.001 |  |  |  | .02 |
| <25 | 17 (25) | 24 (64.9) |  |  | 12 (30.8) | 14 (60.9) |  |
| >25 | 51 (75) | 13 (35.1) |  |  | 27 (69.2) | 9 (39.1) |  |
| NA | 27 | 23 |  |  | 8 | 8 |  |
| PA (IU/L) |  |  | <.001 |  |  |  | <.001 |
| <135 | 22 (52.4) | 31 (86.1) |  |  | 19 (65.5) | 15 (83.3) |  |
| >135 | 20 (47.6) | 5 (13.9) |  |  | 10 (34.5) | 3 (16.7) |  |
| NA | 53 | 24 |  |  | 18 | 13 |  |
| PL (IU/L) |  |  | <.001 |  |  |  | .02 |
| <60 | 18 (43.9) | 29 (85.3) |  |  | 13 (48.1) | 15 (88.2) |  |
| >60 | 23 (56.1) | 5 (14.7) |  |  | 14 (51.9) | 2 (11.8) |  |
| NA | 54 | 26 |  |  | 20 | 14 |  |
| Note. —Data are pancreatic nodules and data in parentheses are percentages. SPL= solid pancreatic lesion, MSPL = malignant solid pancreatic lesion, BSPL = benign solid pancreatic lesion, CEA = carcinoembryonic antigen, CA 19-9 = carbohydrate antigen 19-9, CA 125 = cancer antigen 125, PA = pancreatic amylase, PL = pancreatic lipase, NA = not available. | | | | | | | |
